# Lithium Inhibits GSK3β and Augments GluN2A Receptor Expression in the Prefrontal Cortex

**DOI:** 10.3389/fncel.2018.00016

**Published:** 2018-02-01

**Authors:** Sarah A. Monaco, Brielle R. Ferguson, Wen-Jun Gao

**Affiliations:** Department of Neurobiology and Anatomy, Drexel University College of Medicine, Philadelphia, PA, United States

**Keywords:** glycogen synthase kinase 3β, β-catenin, NMDA receptors, lithium chloride, prefrontal cortex, psychiatric disorders

## Abstract

Glycogen synthase kinase 3β (GSK3β) is a highly conserved serine/threonine kinase that has been implicated in both psychiatric and neurodegenerative diseases including schizophrenia, bipolar disorder, and Alzheimer's disease; therefore regulating its activity has become an important strategy for treatment of cognitive impairments in these disorders. This study examines the effects of lithium on GSK3β and its interaction with β-catenin and NMDA receptors within the prefrontal cortex. Lithium, a clinically relevant drug commonly prescribed as a mood stabilizer for psychiatric disorders, significantly increased levels of phosphorylated GSK3β serine 9, an inhibitory phosphorylation site, and decreased β-catenin ser33/37/thr41 phosphorylation *in vitro*, indicating GSK3β inhibition and reduced β-catenin degradation. GluN2A subunit levels were concurrently increased following lithium treatment. Similar alterations were also demonstrated *in vivo;* lithium administration increased GSK3β serine 9 phosphorylation and GluN2A levels, suggesting a reduced GSK3β activity and augmented GluN2A expression. Correspondingly, we observed that the amplitudes of evoked GluN2A-mediated excitatory postsynaptic currents in mPFC pyramidal neurons were significantly increased following lithium administration. Our data suggest that GSK3β activity negatively regulates GluN2A expression, likely by mediating upstream β-catenin phosphorylation, in prefrontal cortical neurons. Furthermore, our biochemical and electrophysiological experiments demonstrate that lithium mediates a specific increase in GluN2A subunit expression, ultimately augmenting GluN2A-mediated currents in the prefrontal cortex.

## Introduction

Glycogen synthase kinase 3β (GSK3β) plays an important role in both psychiatric and neurodegenerative disorders (Hur and Zhou, 2010; Eldar-Finkelman and Martinez, 2011; King et al., 2014; Beurel et al., 2015), likely due to its importance in synaptic plasticity (Hooper et al., 2007; Peineau et al., 2007, 2009; Zhu et al., 2007; Bradley et al., 2012; Beurel et al., 2015; Xing et al., 2016). In fact, abnormal GSK3β activity has been linked to schizophrenia, bipolar disorder, autism spectrum disorders, as well as Alzheimer's disease in which hyperactivity is commonly reported (Emamian et al., 2004; Hur and Zhou, 2010; Eldar-Finkelman and Martinez, 2011; Beurel et al., 2015). Hyperactive GSK3β is conjectured to be neuropathological; therefore inhibiting its activity may offer as a promising therapeutic option for treating cognitive impairments (King et al., 2014). Although GSK3β inhibition emerges as a hopeful avenue for intervention, and several available pharmacological agents appear to be successful in mitigating symptoms in psychiatric disease by modulating GSK3β activity, how these inhibitors ultimately affect synaptic function and improve cognitive function remains unknown. In particular, lithium has been prescribed as a primary treatment of bipolar disorder, but its mechanisms of action are still ambiguous.

GSK3β regulates a broad range of substrates and is involved in several signal transduction pathways, with the Wnt/β-catenin signaling pathway being the most canonical and well-studied. The Wnt pathway regulates β-catenin stability and ultimately gene expression through the regulation of a degradation complex. The degradation complex consists of five key proteins including: Axin, APC (adenomatous polyposis coli), CK (casein kinase), GSK3β, and β-catenin (Nakamura et al., 1998; Kikuchi, 1999; Kadoya et al., 2000; Hur and Zhou, 2010). GSK3β phosphorylates β-catenin, Axin, and APC. The phosphorylation of APC and Axin enhances binding to β-catenin as well as stabilization (Kikuchi, 1999; Kadoya et al., 2000). In the absence of Wnt, β-catenin is bound to the active degradation complex and phosphorylated by GSK3β. This phosphorylation event tags β-catenin, resulting in ubiquitination and degradation by proteasomes. However, in the presence of Wnt, β-catenin dissociates from the complex, no longer tagged for degradation, and begins to accumulate in the cytoplasm (Nakamura et al., 1998; Kikuchi, 1999; Kadoya et al., 2000; Hur and Zhou, 2010).

GSK3β inhibition has been demonstrated to reduce β-catenin phosphorylation and increase β-catenin stabilization, thereby affecting gene expression and proliferation during early neurodevelopment (Mao et al., 2009). GSK3β complexes have been reported to localize at dendritic synapses, but their function remains unknown (Brandon and Sawa, 2011). β-catenin, like GSK3β, is also localized within dendrites and interacts with NMDARs (Husi et al., 2000; Okabe et al., 2003; Al-Hallaq et al., 2007; Li and Gao, 2011). Following neuronal depolarization, β-catenin localization shifts from the dendritic shaft to spines, and can impact synaptic size as well as strength (Husi et al., 2000; Okabe et al., 2003; Li and Gao, 2011). Accordingly, β-catenin is suggested to be one component of the NMDA multiprotein complex that is present at the postsynaptic density and is possibly involved in regulating spine morphology (Okabe et al., 2003). Furthermore, β-catenin demonstrates subunit specificity, co-localizing to a greater extent with GluN2A compared to GluN2B (Al-Hallaq et al., 2007). Because GSK3β, β-catenin, and NMDARs all localize within dendritic synapses and β-catenin has been reported to interact with both proteins, we investigated whether the GSK3β/β-catenin signaling pathway regulates NMDAR subunits, particularly GluN2A. Therefore, we investigated how GSK3β inhibition affected its common downstream target, β-catenin as well as the effects on NMDAR expression.

We hypothesize that GSK3β regulates NMDA receptor subunit expression by regulating catabolism of the transcription factor, β-catenin, in prefrontal cortical neurons. To address this, we first utilized cultured primary prefrontal cortical neurons to explore how lithium, a direct inhibitor of GSK3β, affects GSK3β kinase activity, β-catenin, and NMDA receptor expression. Utilizing the most efficacious dose and time point from our *in vitro* experiments; we administered lithium *in vivo* to investigate if similar protein and corresponding physiological changes occurred. We centralized our studies around NMDA receptors because these receptors are crucial in prefrontal cortex-dependent cognitive function (Krystal et al., 1994; Malhotra et al., 1996; Newcomer et al., 1999; Hetem et al., 2000; Wang, 2001; Parwani et al., 2005; Tsukada et al., 2005; Cui et al., 2011; Gilmartin et al., 2013; Wang et al., 2013). Additionally, NMDA receptor disruption has been well characterized in animal models of schizophrenia and other neurological disorders, demonstrating a paramount role that they play in pathology (Monaco et al., 2015).

## Materials and methods

Animal procedures used were in accordance with the National Institutes of Health (NIH, USA) animal guidelines and the experimental protocols (#20280) were approved by the Institutional Animal Care and Use Committee at Drexel University College of Medicine. Timed pregnant (TP16) and male (200–250 g) Sprague-Dawley (SD) rats were purchased from Charles River Laboratories (Wilmington, MA, USA).

### Primary prefrontal neuronal culture

Preparation of rat prefrontal cultures was modified from previously described methods (Wang et al., 2003). Briefly, the PFC was dissected from Embryonic day 18 (E18) rat embryos. Cells were dissociated using papain (45-min incubation at 37°C, gently shaking every 15 min) and triturated through Pasteur pipettes. Neurons were plated directly onto wells coated with poly-D-lysine hydrobromide (50 μg/μl) in serum-free media (Neurobasal medium, B27 supplement, 20% glucose, 0.5% glutamine, 1% penicillin/streptomycin), supplemented with 5% horse serum at a density of 400,000 cells/ml. After 3 h, allowing time for cell adhesion, half of the media was changed with warmed serum-free media. On day *in vitro* (DIV) four, half of the media was replaced with fresh serum-free media. Cultures were maintained at 37°C for at least 2 weeks before use.

### Pharmacological treatment

Drug preparation and dose was modified from previously described methods (Rao et al., 2005; Chen et al., 2007). GSK3β inhibitor, lithium chloride (Abcam, Cambridge, MA), was dissolved as a concentrated stock in water and stored at −20°C. Stocks were thawed and appropriately diluted immediately before use. Cultured prefrontal cortical neurons (14–17 DIV) were treated with lithium chloride (5 mM for 4 and 24 h).

A previous study conducted by Rao and colleagues (2005) published a dose-response curve (0, 10, 20, and 500 μM; 1, 5, 10, and 20 mM) demonstrating lithium's effect on GSK3β activity, comparing phosphorylation of GSK3β serine 9 to total protein levels. Lithium chloride treatment in the dose range of 5–10 mM was shown to inhibit the enzymatic activity of GSK3β by more than 80%. A 5 mM dose of lithium was sufficient to reduce GSK3 kinase activity, while concurrently increasing total β-catenin levels starting at 6 h following treatment (Rao et al., 2005).

For *in vivo* administration, lithium chloride was initially dissolved in ddH_2_O a day before use at a concentration of 1 M and stored overnight at 4°C. A 50 mg/kg dose was administered in the intraperitoneal cavity (i.p.) of male SD rats weighing between 200 and 250 g. Control animals were injected with an equal volume of saline. These doses were chosen because serum levels have been reported to fall within the therapeutic range of drug in patients (0.8–1.0 mmol/L) and higher doses were demonstrated to produce adverse side effects (Yang et al., 2001; Gould et al., 2004; Nejadkey et al., 2006; Hillert et al., 2012; Albayrak et al., 2013). After 24 h, PFC tissue was collected. Briefly, rats were lethally injected with 0.2 mg/kg of Euthasol. Once unresponsive to toe- and tail-pinch, rats were transcardially perfused with ice-cold perfusion buffer. Following perfusion, the PFC was dissected and immediately stored on dry ice. Tissue was stored at −80°C until use for Western blotting.

### Cell collection

Plates were set on ice, and all media was removed using vacuum suction. Wells were washed twice with cold 0.1 M PBS, all solution was removed, 50 μl of RIPA buffer (65 mM Tris pH 7.4, 154 mM NaCl, 50 mM Na_4_P_2_O_7_, 25 mM glycerol-2-phosphate, 1 mM EDTA, 0.1% SDS, 0.1% TritonX-100, 0.25% sodium deoxycholate, 500 mM NaF, 100 mM Na_3_VO_4_, 1 mg/ml leupeptin, 1 mg/ml aprotinin, 100 mM PMSF, and 1 mg/ml pepstatin A) was added to each well, and incubated for 15 min at 4°C with gentle rocking. The RIPA buffer contained the following inhibitors: sodium pyrophosphate tetrabasic (Na_4_P_2_O_7_, inhibits Ser/Thr phosphatases), ethylenediaminetetraacetic acid (EDTA, chelates cations), sodium fluoride (NaF, inhibits Ser/Thr and acidic phosphatases), sodium orthovanadate (Na_3_VO_4_, inhibits Tyr, and alkaline phosphatases), leupeptin (inhibits serine and cysteine proteases), aprotinin (inhibits serine proteases), PMSF (inhibits serine proteases), and pepstatin A (inhibits aspartic acid proteases). Adherent cells were collected with a cell scraper, and the cell lysate was transferred to a microcentrifuge tube (combining wells of the same treatment). Lysates were homogenized with a mortar and pestle for 20 strokes and then incubated for 45 min on ice. After incubation, samples were centrifuged at 10,000 g for 10 min at 4°C, pellets discarded, and the supernatant collected and stored at −20°C. Western samples (20 μg/15 μl) were prepared, heated at 95°C for 5 min, cooled to room temperature, and stored at −20°C.

### Prefrontal tissue processing

Tissue from the medial PFC (mPFC) was thawed, weighed, and homogenized in 9 volumes of RIPA buffer containing inhibitors using a mortar and pestle. Lysates were incubated on ice for 30 min. After incubation, samples were centrifuged at 10,000 g for 15 min at 4°C, and the supernatant was collected and stored at −20°C. Western samples (15 μg/15 μl) were prepared, heated at 95°C for 5 min, cooled to room temperature, and stored at −20°C.

### SDS-PAGE and western blot

Whole cell fraction samples (20 μg/15 μl *in vitro*; 15 μg/15 μl *in vivo*) were run on acrylamide gels comprised of a 3.0% acrylamide stacking gel on top of a 7.5% acrylamide separating gel. Proteins were transferred to a polyvinylidene fluoride (PVDF) membrane (Millipore, Immobilon-P membrane) with a 5% methanol transfer buffer at 100 V for 1 h at 4°C. Membranes were trimmed, rinsed in Tris-buffered saline with 1% Tween-20 (TBST) and blocked for 1 h with 5% milk in TBST. Then, primary antibodies were incubated overnight at 4°C with agitation. The following antibodies were applied at the approximate concentrations listed: GSK3β (Cell Signaling Technology, #9315S, RRID:AB_490890, 1:20,000 for cultured cells; 1:10,000 for *in vivo* tissue), pGSK3β serine 9 (Cell Signaling Technology, #9336S, RRID:AB_331405, 1:10,000 for cultured cells; 1:5,000 for *in vivo* tissue), GluN1 (Millipore, #32-0500, RRID:AB_2533060, 1:1,000), GluN2A (Millipore, #04-901, RRID:AB_11213445, 1:1,000), GluN3A (Millipore, #07-356, RRID:AB_2112620, 1:1,000), GluN2B (Millipore, #05-920, RRID:AB_417391, 1:1,000), β-catenin (Cell Signaling Technology, #2698S, RRID:AB_1030945, 1:5,000) and pβ-cateninS33/37/T41 (Cell Signaling Technology, #9561S, RRID:AB_331729, 1:1,000). β-actin (Sigma-Aldrich, A5316, RRID:AB_476743, 1:100,000) was used as a loading control. After primary antibody incubation, membranes were rinsed with TBST three times for 20 min, blocked for 1 h with 5% milk in TBST, incubated with secondary antibody (anti-mouse, Vector Laboratories, #PI-2000, RRID:AB_2336177, 1:2,000; anti-rabbit, Vector Laboratories, #PI-1000, RRID:AB_2336198, 1:2,000) for 1 h, and rinsed with TBST three times for 20 min at room temperature with agitation. Membranes were developed with ECL detection kit (GE Healthcare Bio-Sciences, Piscataway, NJ). After developing, membranes were rinsed with TBST for 5 min and stripped with Restore Western Blot Stripping Buffer (ThermoFisher Scientific, Waltham, MA) for an additional 15–30 min, depending on the antibody intensity. Membranes were then rinsed with TBST 3 times for 10 min. To probe the membrane for additional proteins the entire process, the described sequence was repeated from primary antibody incubation; membranes were blocked with 5% milk in TBST for 1 h and then incubated with primary antibody overnight at 4°C with agitation. Protein expression was determined using Bio-Sciences, Piscataway, NJ ImageJ64 software.

### Whole-cell electrophysiology

Male SD rats were injected with Lithium (50 mg/kg, i.p.) or an equivalent dose of physiological saline, and were sacrificed for slice collection after 24 h. Animals were perfused with ice-cold sucrose buffer, decapitated, and the brain was extracted. The mPFC was dissected in ice-cold sucrose solution and glued to a block in a vibratome bath containing the same solution (Leica VT1200S, Leica Microsystems, Buffalo Grove, IL). Slices containing the mPFC (300 μm) were collected and transferred to a slice chamber containing oxygenated ACSF that was bubbled continuously with 95% O_2_ and 5% CO_2_ and incubated at 37 degrees C for 45 min. Neurons were visualized with infrared differential interference video microscopy.

All experiments were conducted with the Axon MultiClamp 700B amplifier, and data were acquired using pCLAMP 9.2 software (Molecular Devices, Sunnyvale, CA). Recordings were obtained using a Cesium (Cs^+^)-based intracellular solution (in mM: 110 D-gluconic acid, 110 CsOH, 10 CsCl_2_, 1 EGTA, 1 CaCl_2_, 5 QX-314, 1 ATP-Mg, 10 HEPES, at pH 7.3, adjusted with CsOH) and in the presence of picrotoxin (50 μM), DNQX (20 μM), and Ro-25-6981 (0.5 μM), to block GABA_A_, AMPA, and GluN2B-containing NMDA receptor-mediated currents respectively (Wang et al., 2008; Wang and Gao, 2009). Layer II/III was stimulated with single pulses to evoke excitatory postsynaptic currents (eEPSCs) in Layer V pyramidal neurons. For electrophysiological data, Clampfit 9.2 was used to determine the amplitude of eEPSCs.

### Data analysis

Western blot protein expression was determined using the ImageJ64 software. Each sample was taken as a ratio to the loading control (e.g., NMDAR subtype to β-actin). In the case of phosphorylation, each sample was taken as a ratio of its respective total protein levels (e.g., pGSK3β ser9 to GSK3β). Total protein in the text refers to an antibody that tags the target protein without specificity for a particular post-translational modification such as phosphorylation, accounting for cellular levels of that protein under all conditions. For example, total GSK3β would encompass phosphorylated as well as unphosphorylated levels. *In vitro* data included 3 pregnant dams, with approximately 10–12 rat embryos each, from which primary neuronal cultures were generated. Each treatment group included 5–6 animals for all *in vivo* data. To reduce intra-blot variability, each tissue sample was run 4 times to obtain an average. For whole-cell electrophysiology, the control group includes 14–15 cells and the lithium-treated group contains 16–17 cells. *In vitro* data were analyzed with a two-way ANOVA, while *in vivo* data were analyzed with an independent *T*-test using SPSS statistics 24 (IBM Statistics, Armonk, New York). All data underwent Shapiro-Wilk normality tests and Levene's Test of Equality of Error Variances when appropriate. The data without normal distribution were analyzed with a non-parametric Mann-Whitney *U*-test using SPSS. All data was presented as a mean ± standard error (S.E). Level for significance was set at *p* ≤ 0.05 for all comparisons.

## Results

### Lithium increased pGSK3β ser9, but not total GSK3β levels *in vitro* and *in vivo*

To replicate prior research findings (Rao et al., 2005; Chen et al., 2007), we utilized lithium at a previously described dose to pharmacologically inhibit GSK3β, comparing serine 9 phosphorylation levels to total levels of GSK3β as an indication of inhibition. Primary prefrontal cortical cultures were treated with vehicle or LiCl (5 mM) for two time points, 4 and 24 h. Western blot analysis was used to determine protein expression levels of GSK3β and pGSK3β ser9, which is an inhibitory phosphorylation site.

**Figure 1:**
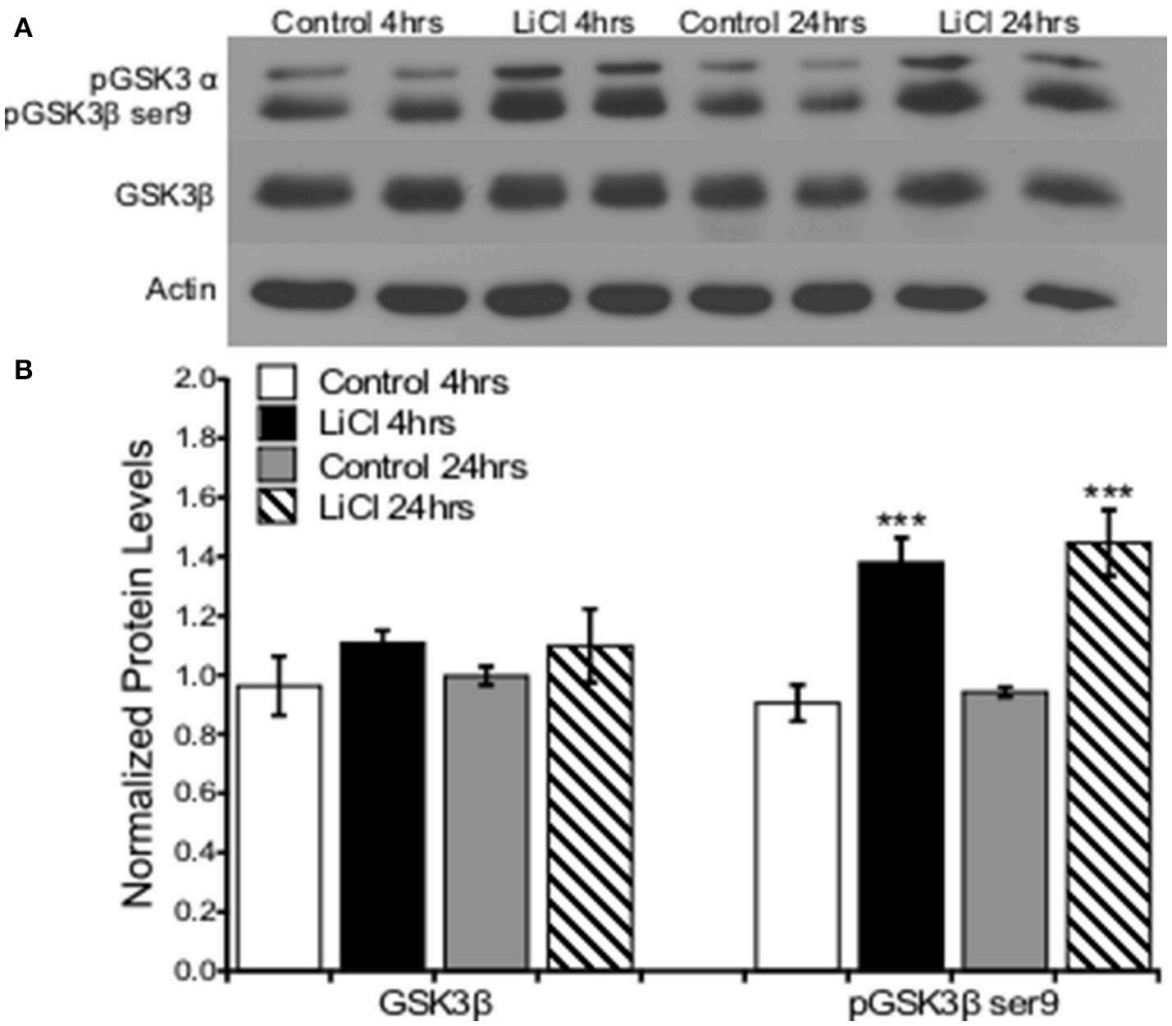
Lithium increased pGSK3β ser9, but not total GSK3β levels in cultured prefrontal cortical neurons. **(A)**. Representative images of Western blots. **(B)**. The summary histogram shows GSK3βser9 phosphorylation increased (*p* = 0.000), while total levels remained unaltered following lithium treatment (*p* = 0.180). Total protein levels were normalized to actin, while phosphorylated protein levels were normalized to total protein. Data are presented as a mean ± standard error (S.E.). Level for significance was set at *p* ≤ 0.05 for all comparisons, ^***^P ≤ 0.001.

A two-way ANOVA was conducted to determine the effects of treatment group and time on total GSKβ as well as GSK3β serine 9 phosphorylation protein levels. There was no significant effect of treatment group, *F*_(1, 8)_ = 2.163, *p* = 0.180, or time, *F*_(1, 8)_ = 0.020, *p* = 0.891, on total GSK3β protein levels. There was no significant interaction between treatment group and time point on GSK3β protein levels, *F*_(1, 8)_ = 0.069, *p* = 0.799. Lithium treatment significantly increased GSK3β ser9 phosphorylation protein levels, *F*_(1, 8)_ = 40.593, *p* = 0.000. There was no significant effect of time on GSK3β ser9 phosphorylation, *F*_(1, 8)_ = 0.467, *p* = 0.514. There was no significant interaction between treatment group and time point on GSK3β ser9 phosphorylation protein levels, *F*_(1, 8)_ = 0.037, *p* = 0.853 (Figure 1).

**Figure 2:**
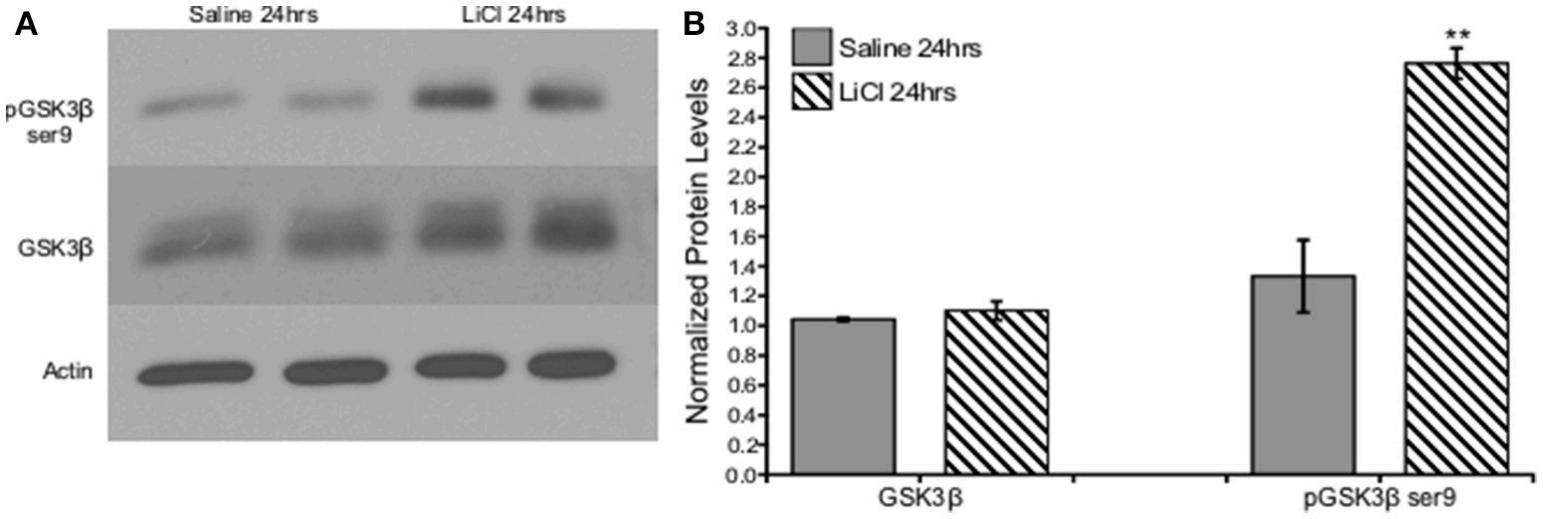
Lithium increased pGSK3β ser9, but not total GSK3β levels *in vivo*. **(A)**. Representative images of Western blots. **(B)**. Summary histogram shows GSK3βser9 phosphorylation significantly increased (*p* = 0.004), while total levels remained unaltered following lithium treatment (*p* = 0.394). Total protein levels were normalized to actin, while phosphorylated protein levels were normalized to total protein. Data are presented as a mean ± standard error (S.E.). Level for significance was set at *p* ≤ 0.05 for all comparisons, ^**^P ≤ 0.01.

A critical question of whether these findings derived from cell culture could also be replicated *in vivo* led us to investigate the effect of lithium on GSK3β in an adult rodent. A 50 mg/kg dose of lithium was administered to male SD rats and PFC tissue was collected after 24 h. A Mann-Whitney *U*-test was run to determine if there were differences in total and phosphorylated GSK3β serine 9 protein levels between saline and lithium-treated animals. Lithium treatment had no effect on GSK3β protein levels, *U* = 12, *z* = −0.961, *p* = 0.394. However, lithium administration significantly increased GSK3β serine 9 phosphorylation, *U* = 35, *z* = 2.722, *p* = 0.004 (Figure 2). Therefore, we conclude that our *in vitro* findings were replicated *in vivo*, demonstrating that lithium increased GSK3β ser9 phosphorylation, in agreement with previous reports (Rao et al., 2005; Chen et al., 2007).

### Lithium decreased β-catenin ser33/37/thr41 phosphorylation levels *in vitro*

Next, we investigated the effects of GSK3β inhibition on downstream cellular targets. We focused on the role GSK3β plays in regulating β-catenin because of previous findings, which demonstrated that GSK3β inhibition reduced β-catenin phosphorylation and therefore stabilized β-catenin levels. Furthermore, β-catenin stabilization or GSK3β inhibition was able to reverse schizophrenia- and depression-like behaviors as well as the associated cellular phenotype. Collectively, these data demonstrate the importance of GSK3β/β-catenin as a potential pathway involved in the etiology of psychiatric disorders (Mao et al., 2009).

**Figure 3:**
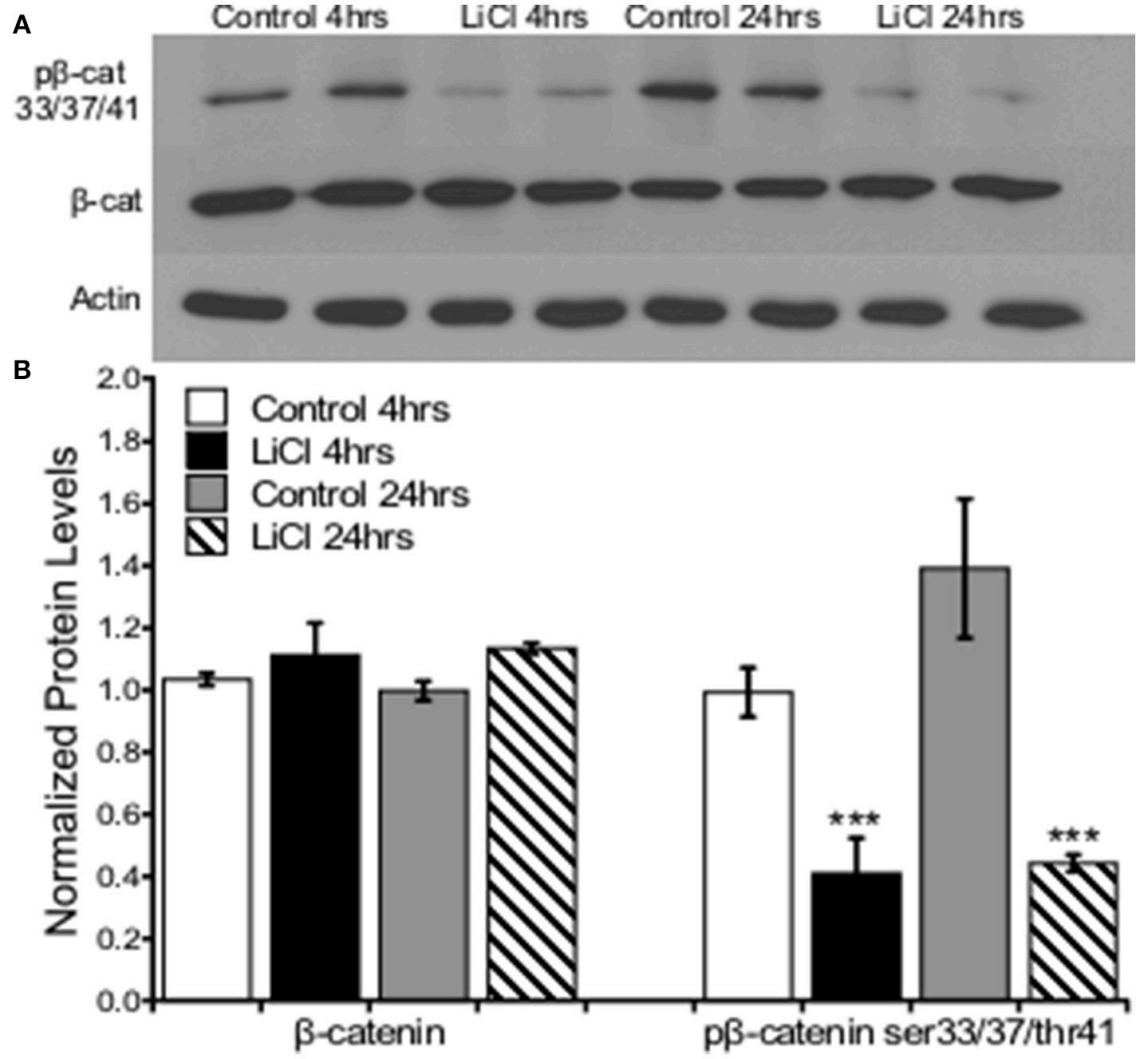
Lithium decreased pβ-catenin ser33/37/thr41, but not total β-catenin levels in prefrontal cortical neurons. **(A)** Representative images of Western blots. **(B)** Summary histogram shows decreased β-catenin ser33/37/thr41 phosphorylation at both time points (*p* = 0.000) in prefrontal cortical cultures following lithium treatment. No changes in total β-catenin protein levels were observed (*p* = 0.090). Total protein levels were normalized to actin, while phosphorylated protein levels were normalized to total protein. Data are presented as a mean ± standard error (S.E.). Level for significance was set at *p* ≤ 0.05 for all comparisons, ^***^P ≤ 0.001.

Prefrontal cortical cultures were treated with vehicle or LiCl (5 mM) for two time points, 4 or 24h, and Western blot analysis was used to determine protein expression levels of β-catenin and pβ-catenin ser33/37/thr41, the GSK3β target regulatory site. A two-way ANOVA was conducted to determine the effects of treatment group and time on total β-catenin as well as β-catenin ser33/37thr41 phosphorylation protein levels. There was no significant effect of treatment group, *F*_(1, 8)_ = 3.710, *p* = 0.090, or time, *F*_(1, 8)_ = 0.017, *p* = 0.899, on total β-catenin protein levels. There was no significant interaction between treatment group and time point on β-catenin protein levels, *F*_(1, 8)_ = 0.269, *p* = 0.618. Lithium treatment significantly decreased β-catenin ser33/37thr41 phosphorylation protein levels, *F*_(1, 8)_ = 33.341, *p* = 0.000. There was no significant effect of time on β-catenin ser33/37thr41 phosphorylation, *F*_(1, 8)_ = 2.652, *p* = 0.142. There was no significant interaction between treatment group and time point on β-catenin ser33/37thr41 phosphorylation protein levels, *F*_(1, 8)_ = 1.896, *p* = 0.206 (Figure 3).

**Figure 4:**
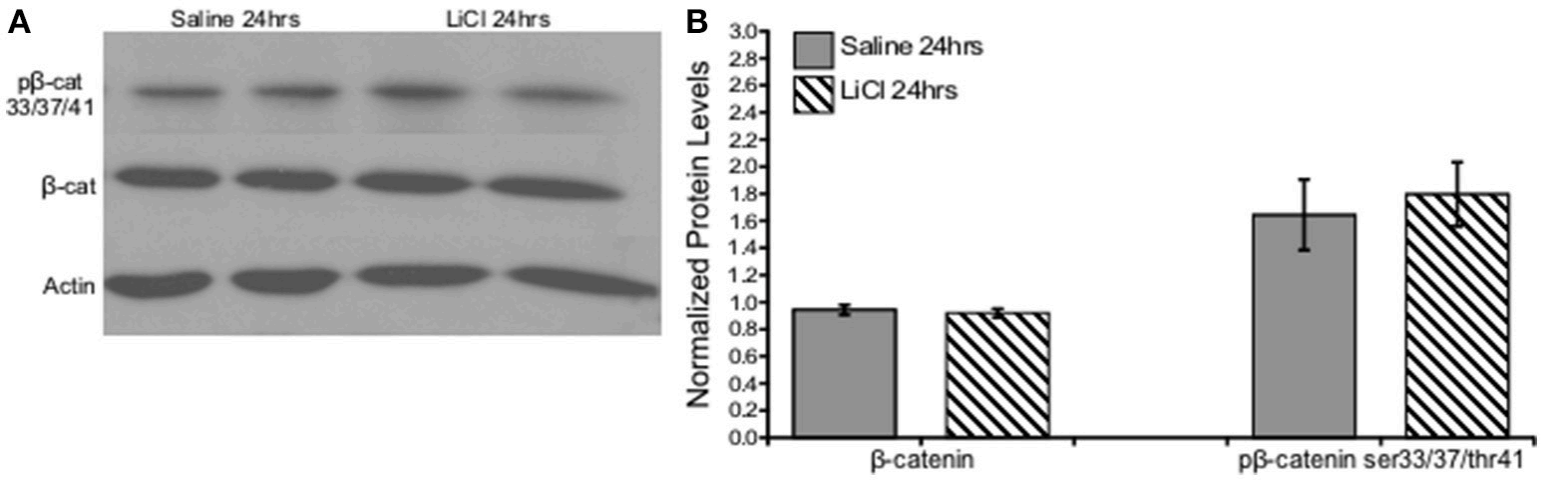
Lithium had no effect on β-catenin or β-catenin ser33/37/thr41 phosphorylation *in vivo*. **(A)** Representative images of Western blots. **(B)** Summary histogram shows no changes in protein level expression of either β-catenin (*p* = 0.616) or β-catenin ser33/37/thr41 phosphorylation (*p* = 0.675) following lithium treatment. Data are presented as a mean ± standard error (S.E.). Level for significance was set at *p* ≤ 0.05 for all comparisons.

An independent-samples *t*-test was run to determine if there were differences in total or phosphorylated β-catenin ser33/37/thr41 protein levels between saline and lithium-treated animals. Lithium administration had no effect on either β-catenin, *t*_(10)_ = 0.518, *p* = 0.616, or β-catenin ser33/37/thr41 phosphorylation, *t*_(10)_ = −0. 431, *p* = 0.675 (Figure 4).

### Lithium increased NMDA expression in a subunit-specific manner *in vitro* and *in vivo*

Because NMDARs are essential for cognitive function and GSK3β has been associated with synaptic plasticity, we therefore investigated how inhibiting GSK3β would affect NMDAR expression. Prefrontal cortical cultures were treated with LiCl (5 mM) for two time points, 4 or 24 h and Western blot analysis was used to determine protein expression levels of NMDAR subunits GluN2A, GluN2B, GluN3A, and GluN1.

**Figure 5:**
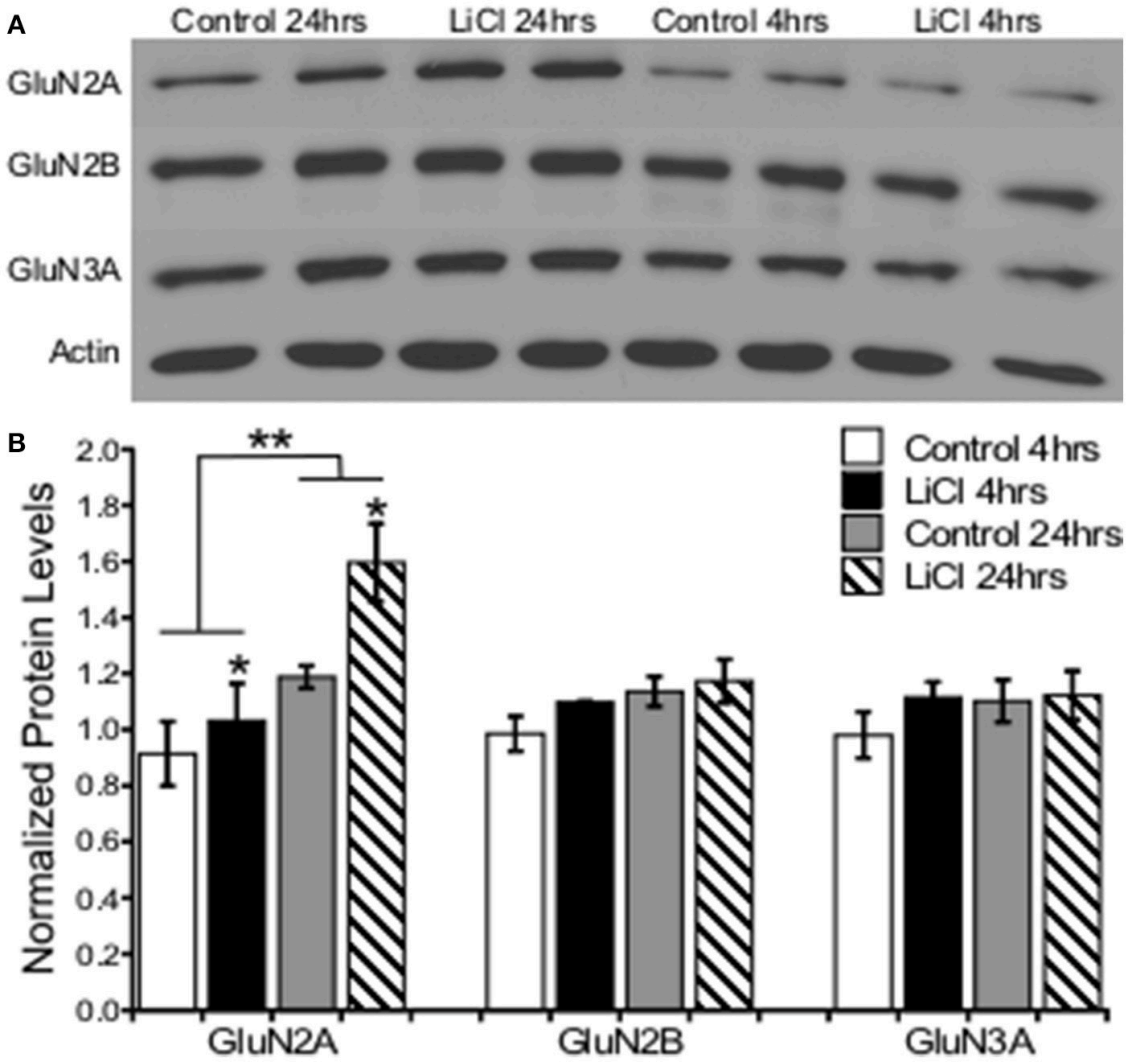
Lithium increased NMDA receptor expression in a subunit-specific manner *in vitro*. **(A)** Representative images of Western blots. **(B)** Summary histogram shows lithium significantly increased GluN2A expression levels (*p* = 0.050), but not GluN2B (*p* = 0.225) or GluN3A (*p* = 0.347). GluN2A was significantly increased at the 24-h time point (*p* = 0.006). Total protein levels were normalized to actin. Data are presented as a mean ± standard error (S.E.). Level for significance was set at *p* ≤ 0.05 for all comparisons, ^*^P ≤ 0.05; ^**^P ≤ 0.01.

A two-way ANOVA was conducted to determine the effects of treatment group and time on NMDARs protein levels. Lithium treatment significantly increased GluN2A protein levels, *F*_(1, 8)_ = 5.343, *p* = 0.050. There was a significant effect of time on GluN2A expression, *F*_(1, 8)_ = 13.559, *p* = 0.006, increasing after 24 h; but no significant interaction between treatment group and time point on GluN2A protein levels, *F*_(1, 8)_ = 1.627, *p* = 0.238. There was no significant effect of treatment group, *F*_(1, 8)_ = 1.728, *p* = 0.225, or time, *F*_(1, 8)_ = 4.029, *p* = 0.080, as well as no significant interaction between treatment group and time point on GluN2B protein levels, *F*_(1, 8)_ = 0.437, *p* = 0.527. Additionally, there was also no significant effect of treatment group, *F*_(1, 8)_ = 0.999, *p* = 0.347, or time, *F*_(1, 8)_ = 0.714, *p* = 0.423, as well as no significant interaction between treatment group and time point on GluN3A protein levels, *F*_(1, 8)_ = 0.561, *p* = 0.475 (Figure 5).

**Figure 6:**
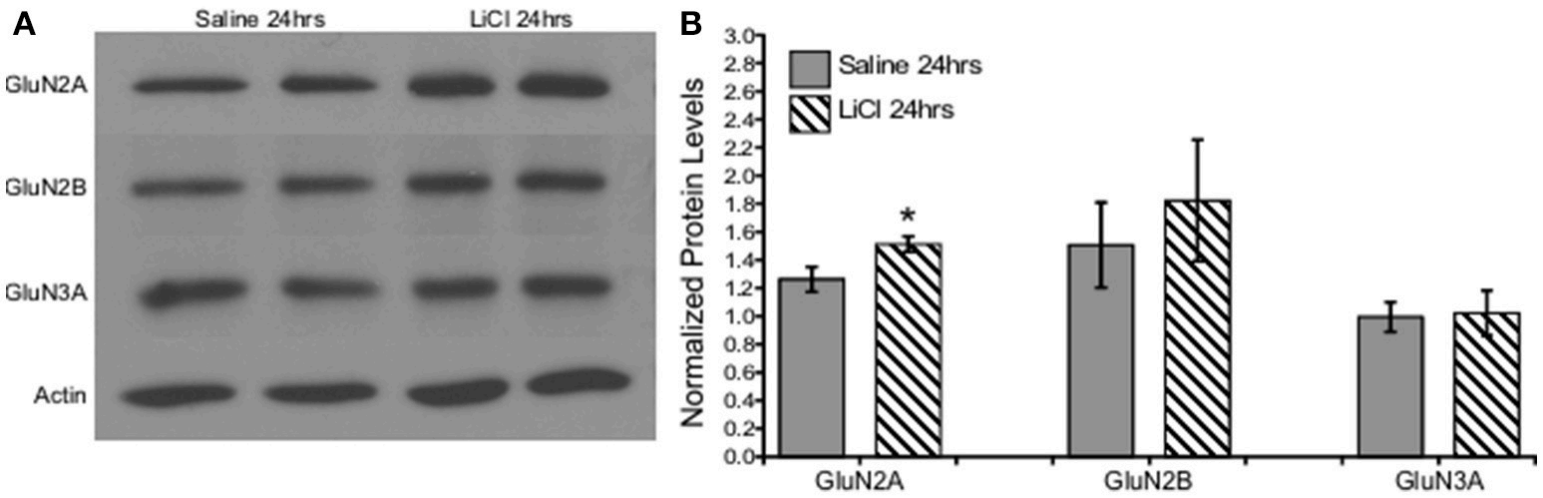
Lithium increased NMDA expression in a subunit-specific manner *in vivo*. **(A)** Representative images of Western blots. **(B)** Summary histogram shows a selective increase in GluN2A expression levels (*p* = 0.038) at 24 h following lithium treatment relative to control. Data are presented as a mean ± standard error (S.E.). Level for significance was set at *p* ≤ 0.05 for all comparisons, ^*^P ≤ 0.05.

**Figure 7:**
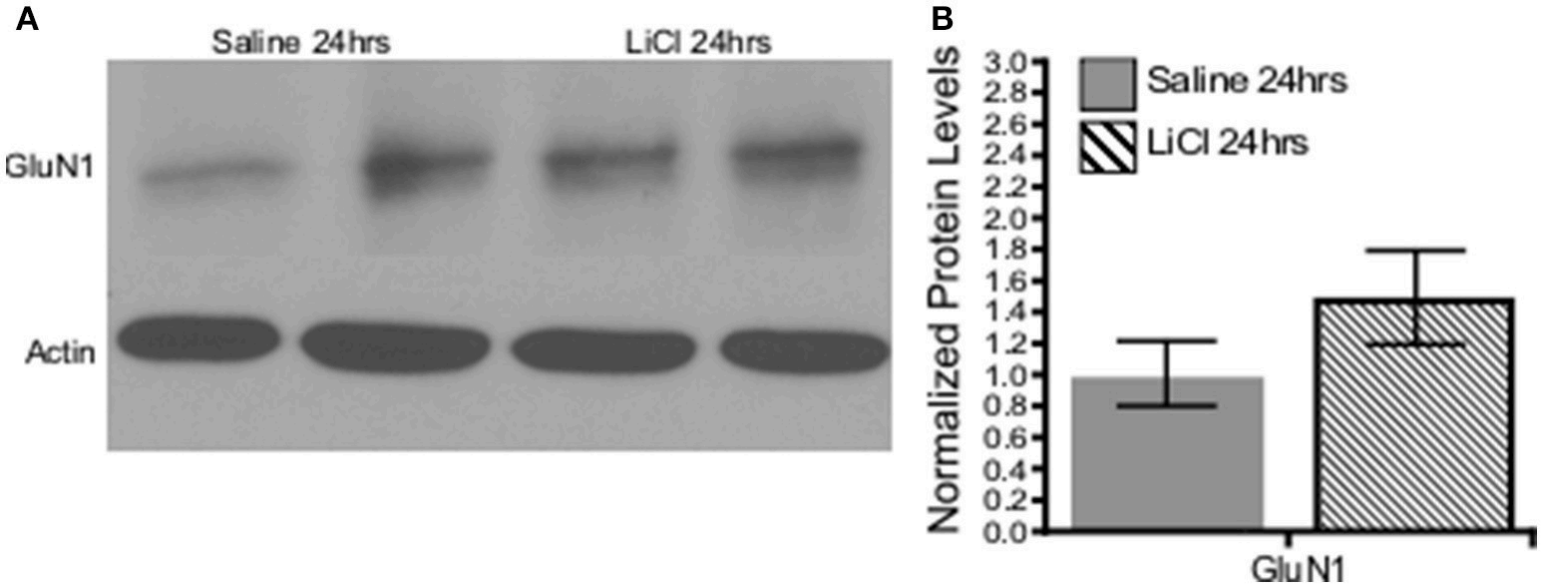
Lithium had no effect on the obligatory GluN1 subunit expression *in vivo*. **(A)** Representative images of Western blots. **(B)** Summary histogram shows no difference in GluN1 expression levels at 24 h following lithium treatment relative to control (*p* = 0.219, *n* = 5 for all). Data are presented as a mean ± standard error (S.E.). Level for significance was set at *p* ≤ 0.05 for all comparisons.

A combination of Mann-Whitney *U*-test and independent-samples *t*-test was run to determine if there were differences in NMDAR subunit expression between saline and lithium-treated animals. Lithium administration had no effect on GluN2B *U* = 24, *z* = 0.961 *p* = 0.394, GluN3A *t*_(10)_ = −0.151, *p* = 0.883, or GluN1 *t*_(8)_ = −1.333, *p* = 0.219, protein levels compared to saline-treated animals (Figures 6, 7). However, lithium administration selectively increased total GluN2A protein levels, *t*_(10)_ = −2.387, *p* = 0.038 (Figure 6). These results indicate that lithium treatment affects NMDA protein expression in a subunit-specific and time-dependent manner and these effects are possibly due to regulation via β-catenin.

### Lithium augments GluN2A-evoked excitatory postsynaptic current amplitude *in vivo*

**Figure 8:**
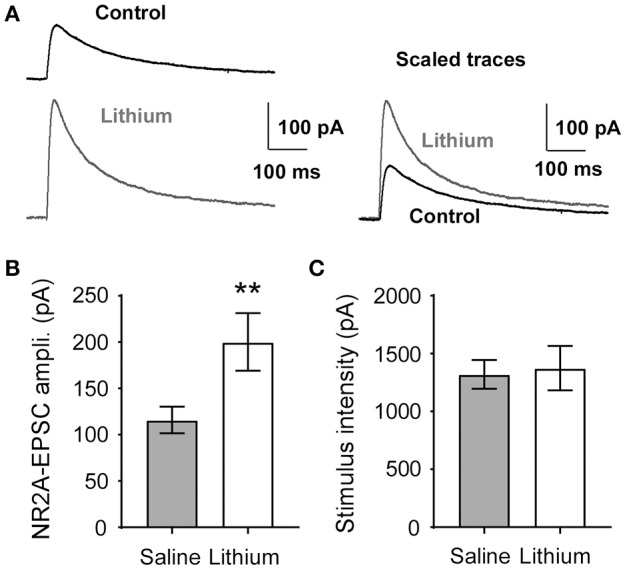
Lithium augments evoked GluN2A-mediated EPSC amplitude. **(A)** Sample traces of GluN2A-evoked EPSCs recorded from the saline control and lithium-treated layer 5 pyramidal neurons in the mPFC. **(B)** The amplitude of GluN2A-EPSCs was significantly increased in lithium-treated rats (*p* = 0.009, *n* = 16) compared to control (*n* = 14). **(C)**, Stimulus intensity required to evoke GluN2A-mediated current in the mPFC is not different between control and lithium-treated rats (*p* = 0.822, saline *n* = 15, lithium *n* = 17). Data are presented as a mean ± standard error (S.E.). Level for significance was set at *p* ≤ 0.05 for all comparisons, ^**^P ≤ 0.01.

To measure the functional consequences of upregulated GluN2A expression in the prefrontal cortex (PFC), we treated rats with lithium (50 mg/kg) and measured evoked GluN2A-mediated excitatory postsynaptic currents (eEPSCs) in the layer 5 pyramidal neurons of the mPFC. Based on our previous reports (Wang et al., 2008; Wang and Gao, 2009) and others (McQuail et al., 2016), after blocking GluN2B with a selective antagonist such as ifenprodil or Ro-25-6981, the remaining portion of the NMDA receptor-mediated current was mostly mediated by GluN2A subunits in the rat mPFC pyramidal neurons. A Mann-Whitney *U*-test was run to determine if there were differences in GluN2A-mediated evoked EPSCs between saline and lithium-treated animals. Lithium administration increased the amplitude of GluN2A-mediated eEPSCs in mPFC pyramidal neurons *U* = 174, *z* = 2.578, *p* = 0.009, compared to saline (Figures 8A,B). An independent-samples *t*-test was run to determine if the stimulus intensity between saline and lithium were comparable. The reported changes in GluN2A-eEPSCs were not due to differences in stimulation intensity *t*_(30)_ = −0.227, *p* = 0.822 (Figure 8C). Our findings suggest that the increase in GluN2A receptor protein following lithium administration is responsible for the increase in GluN2A-mediated eEPSCs amplitude.

## Discussion

Regulating GSK3β activity is a promising candidate for alleviating cognitive symptoms associated with psychiatric and neurodegenerative diseases, albeit how current pharmacological agents mediate their beneficial properties remains unclear. In this study, we utilized both *in vitro* and *in vivo* approaches to test the hypothesis that GSK3β affects the expression of NMDA receptors in prefrontal neurons by affecting β-catenin availability. We found that GSK3β influenced NMDA receptor expression in a subunit-specific and time-dependent manner. In addition, β-catenin possibly regulates GluN2A expression *in vitro*, which is influenced by upstream GSK3β activity. Lithium was able to effectively regulate GSK3β activity, and β-catenin phosphorylation, in addition to GluN2A subunit expression *in vitro* and *in vivo*. Complimentary findings were validated by whole cell electrophysiology, demonstrated by augmented GluN2A-mediated eEPSCs.

Although our study solely focuses on the effects of lithium and its regulation of GSK3β, lithium has another major target, inositol monophosphatase (IMPase). Inositol and GSK3 are the two major targets of lithium, which are both argued to underlie its effects. The inositol depletion model proposes that lithium inhibits IMPase, depleting the cell of endogenous inositol, thereby blocking the production of inositol 1,4,5 triphosphate (InsP3) and preventing extracellular signaling by this pathway. Albeit some researchers suggest inositol depletion mediates the effects of lithium (Berridge et al., 1989), others argue that GSK3β plays the predominate role in the mechanism of lithium's action (Klein and Melton, 1996; Stambolic et al., 1996). Klein and Melton (1996) reported that lithium directly inhibited GSK3β and suggested that GSK3β was the endogenous target of lithium, stressing less importance on IMPase (Klein and Melton, 1996). Lithium was also found to mimic Wnt signaling by inhibiting GSK3β (Stambolic et al., 1996; Lucas and Salinas, 1997). More recently, InsP3 largely mediated the effects of lithium during early development processes, whereas GSK3 inhibition effected later development (Williams et al., 2002). Because our studies focus on later neurodevelopmental processes, we suggest that lithium is primarily mediating its effect via GSK3β; however, we cannot completely exclude effects induced by inositol, but based on previous literature predict the effects are less dramatic.

### Decreases in β-catenin phosphorylation

Lithium-induced a change in β-catenin phosphorylation, likely due to its potent inhibition of GSK3β. These results are supported by previously reported literature in which a 5 mM dose of lithium was sufficient to reduce GSK3β kinase activity by 80%, increase total β-catenin levels, while concurrently reducing phosphor-Ser37 β-catenin levels (Rao et al., 2005). A decrease in β-catenin phosphorylation at 4 h was observed because phosphorylation is a rapid event. Following GSK3β inhibition, β-catenin phosphorylation is disrupted and readily detectable. Disruption of the Axin/GSK3β/β-catenin complex occurs within minutes, but stabilization of β-catenin takes several hours because new protein must be synthesized and then accumulated within the cytoplasm (Martinez et al., 2006; Dobrowolski and De Robertis, 2012). A lack of change in total β-catenin protein level might be due to nuclear translocation of the transcription factor. β-catenin is likely accumulating in the nucleus and might contribute to the lack of total change *in vitro* and *in vivo*.

The decrease in β-catenin phosphorylation, along with the selective increase in GluN2A-mediated eEPSCs as well as total protein levels, corresponds with previous results demonstrating the interaction between β-catenin/GluN2A but a lack thereof with β-catenin/GluN2B. β-catenin has been demonstrated to localize within dendritic spines, respond to neural activity, interact with NMDARs at the postsynaptic density, and possibly affect spine morphology; therefore, our data suggests GSK3β could also be involved as well.

### GluN2A subunit expression is regulated by GSK3β in a time-dependent manner

Lithium selectively augmented GluN2A expression after 24 h *in vitro*. Changes in GluN2A expression were not observed until 24 h. NMDA expression is the last step in a pathway that requires several hours for completion. Conclusively, our temporal changes are reasonable considering the cellular events and the timescale at which they progress.

Our *in vivo* lithium administration induced similar changes in PFC protein levels as observed in culture. Serine 9 phosphorylation of GSK3β increased, indicating reduced kinase activity and total GluN2A levels were augmented. Additionally, the lithium-induced increase of GluN2A-mediated eEPSC amplitude in mPFC pyramidal neurons might be explained by an increase in postsynaptic GluN2A expression, which is supported by the biochemical *in vitro* and *in vivo* data, demonstrating an increase in total protein expression. Therefore, our data suggest that GSK3β negatively regulates the expression of GluN2A in the PFC.

An LTP study conducted by Zhu and colleagues (2007) in cultured neurons reported similar findings regarding the relationship between GSK3β and NMDA receptors. Induced activation of GSK3β, either pharmacologically or genetically, led to decreased GluN2A/B expression that could then be mitigated by the simultaneous inhibition of the kinase. This study demonstrates the negative regulation that GSK3β imparts over GluN2A and GluN2B (Zhu et al., 2007). Although we did not observe GluN2B changes, nonetheless these data confirm that GSK3β influences GluN2A expression in an inhibitory fashion and consequently affects the GluN2B/GluN2A ratio, which could, in turn, alter synaptic function and plasticity (Monaco et al., 2015).

Specifically, PFC persistent neuronal firing is the foundation of working memory with NMDA receptor activity playing a substantial role in this process (Wang, 2001; Gilmartin et al., 2013). NMDA antagonism in conscious behaving monkeys impairs prefrontal-dependent working memory (Tsukada et al., 2005) and induces cognitive impairments in healthy human subjects (Krystal et al., 1994; Malhotra et al., 1996; Newcomer et al., 1999; Hetem et al., 2000; Parwani et al., 2005), demonstrating a parallel between NMDA hypofunctioning and cognition deficits. Additionally, antagonizing GluN2B and GluN2A-containing NMDA receptors impairs persistent activity in prefrontal cortical neurons, whereas GluN2B overexpression leads to enhanced LTP and performance on working memory tasks (Cui et al., 2011; Wang et al., 2013).

NMDARs and PFC-dependent cognition also share common ties to the pathology observed in schizophrenia. NMDA receptor disruption has been well characterized in animal models of schizophrenia, demonstrating the paramount role these receptors play in disease. Mutations in high-risk gene candidates for schizophrenia such as neuregulin 1, DISC1, and dysbindin have all been demonstrated to disturb NMDA receptor regulation (Geddes et al., 2011; Karlsgodt et al., 2011; Ma et al., 2013). Additionally, NMDA antagonism has been demonstrated to induce psychosis in normal individuals and exacerbate symptoms in patients with schizophrenia (Javitt and Zukin, 1991). Cognitive impairments, particularly working memory deficits, are a core feature of schizophrenia (Lett et al., 2013).

Surmounting evidence links NMDA receptor hypofunctioning in the PFC, particularly of GluN2A and GluN2B-containing receptors, as an underlying pathological origin of cognitive impairments (Wang et al., 2013; Monaco et al., 2015). Considering that GluN2A is necessary for PFC-dependent cognition as well as GABAergic maturation and maintenance, targeting GSK3β offers as a promising avenue for treating a disorder characterized by these deficits (Kinney et al., 2006; Wang et al., 2013). Indeed, a recent research article highlights the importance of GluN2A in PFC-dependent working memory demonstrating that working memory largely depends on GluN2A, which in turn mediates a majority of the NMDA currents on layer 2/3 pyramidal neurons (McQuail et al., 2016). Additionally, activating GluN2A-NMDA currents was reported to augment working memory in aged rodents (McQuail et al., 2016). In correspondence with our findings, GSK3β inhibition via lithium offers as a pharmacological approach to enhance GluN2A expression as well as evoked GluN2A-current in the PFC of rodents, which has potential to augment working memory performance (King et al., 2014; McQuail et al., 2016).

In summary, pharmacological inhibition of GSK3β with lithium significantly increased pGSK3β ser9 and decreased pβ-catenin ser33/37/thr41, thereby suggesting GSK3β inhibition and reduced β-catenin degradation. Total GluN2A levels concurrently increased after 24 h following lithium, demonstrating preferential upregulation of the GluN2A gene expression. These changes also corresponded with a physiological change in the mPFC, augmenting GluN2A-mediated eESPCs. We speculate this could be due to an increase in GluN2A expression at the postsynaptic density, which could increase the GluN2A-mediated inward current of mPFC pyramidal neurons, leading to increased amplitude of excitatory events. Conclusively, GSK3β activity influences the expression of GluN2A both *in vitro* and *in vivo* with effects possibly mediated by β-catenin phosphorylation in the PFC. It is important to note that a limitation with our study is that our findings are largely correlational since we do not directly test whether lithium inhibits GSK3β kinase activity or selectively manipulate GSK3β in the mPFC, such as the utilization of shRNA. Therefore, our current study has set an essential framework, but future studies are warranted to further untangle the nature of lithium and its possible therapeutic actions of NMDA receptor augmentation in the PFC and the regulation of GSK3β.

## Ethics statement

The authors have read and have abided by the statement of ethical standards for manuscripts submitted to the Frontiers in Cellular Neuroscience. We declare that submitted manuscript does not contain previously published materials and are not under consideration for publication elsewhere. Each author has made a significant scientific contribution to the study, and is familiar with the primary data. All authors listed have read the complete manuscript and have approved submission of the paper. The manuscript is original work without fabrication, fraud, or plagiarism. All authors declare no conflicts of interest.

## Author contributions

SM contributed to the design, acquisition, analysis, and interpretation of the data; BF contributed to collection and interpretation of the electrophysiological data; and W-JG contributed to the conception of the work. All authors contributed to writing and editing the manuscript.

### Conflict of interest statement

The authors declare that the research was conducted in the absence of any commercial or financial relationships that could be construed as a potential conflict of interest.
